# Dual Role of p53 in Innate Antiviral Immunity

**DOI:** 10.3390/v2010298

**Published:** 2010-01-22

**Authors:** Carmen Rivas, Stuart A. Aaronson, Cesar Munoz-Fontela

**Affiliations:** 1 Centro Nacional de Biotecnologia, CSIC, Darwin 3, Campus Universidad Autónoma, Madrid 28049, Spain; E-Mail: crivas@cnb.csic.es (C.R.); 2 Department of Oncological Sciences, Mount Sinai School of Medicine, One Gustave L. Levy Place Box 1130, New York, NY 10029, USA; E-Mail: stuart.aaronson@mssm.edu (S.A.A.)

**Keywords:** p53, interferon, immunity, apoptosis

## Abstract

Tumor suppressor p53 is widely known as ‘the guardian of the genome’ due to its ability to prevent the emergence of transformed cells by the induction of cell cycle arrest and apoptosis. However, recent studies indicate that p53 is also a direct transcriptional target of type I interferons (IFNs) and thus, it is activated by these cytokines upon viral infection. p53 has been shown to contribute to virus-induced apoptosis, therefore dampening the ability of a wide range of viruses to replicate and spread. Interestingly, recent studies also indicate that several IFN-inducible genes such as interferon regulatory factor 9 (IRF9), IRF5, IFN-stimulated gene 15 (ISG15) and toll-like receptor 3 (TLR3) are in fact, p53 direct transcriptional targets. These findings indicate that p53 may play a key role in antiviral innate immunity by both inducing apoptosis in response to viral infection, and enforcing the type I IFN response, and provide a new insight into the evolutionary reasons why many viruses encode p53 antagonistic proteins.

## 1. Introduction

An important host defense mechanism against virus infection, the type I IFNs, are rapidly induced to activate an antiviral state in infected and neighboring uninfected cells. Type I IFNs bind to the type I IFN receptor formed by IFNAR1 and IFNAR2 subunits [1,2] and trigger the activation of the canonical Jak/signal transducer and activator of transcription (STAT) signaling pathway and the trans-activation of antiviral genes, through a mechanism that depends to a great extent on STAT1, STAT2 and IRF9 [3]. These three transcription factors form the trimeric IFN-stimulated gene factor 3 (ISGF3) complex, which is relocated to the nucleus where it binds to the IFN-stimulated response elements (ISREs) in promoters of target genes to activate their transcription. Since type I IFNs stimulate p53 expression and virus infection activates p53 [4], this tumor suppressor has been recently included as a new component of the cellular antiviral defense mechanisms. Studies by us and others in recent years indicate that p53 is in fact, a key player in innate antiviral immunity by both enforcing the type I IFN response upon viral infection, and inducing apoptosis in infected cells [5–9]. Both actions coordinated by this tumor suppressor gene, help to thwart the replication of a wide variety of viruses both *in vitro* and *in vivo* [5–9]. In this review we try to provide a summary of the recent findings that involve p53 in antiviral immunity and that presumably, may help to explain not only why this protein is conserved in invertebrate organisms which do not undergo cancer-related diseases, but also, why this protein is so frequently targeted by viral proteins.

## 2. Regulation of p53 functions

The potent activity of p53 as inducer of cellular apoptosis and cell cycle arrest demands tight control of its function. In normal cells the level of p53 protein is low due to its short half-life [10] regulated mainly by murine double minute 2 (Mdm2) protein. Mdm2 inhibits p53 transactivation and prompts p53 for proteasomal degradation by promoting its ubiquitylation [11,12]. In contrast, p53 is stabilized and activated when, in response to oncogene expression, another tumor suppressor gene, namely ARF, binds to Mdm2 and blocks its ability to inhibit p53 function [13]. Similarly, the nucleolar protein nucleophosmin (NPM) also interacts with Mdm2 and promotes p53 stability and function [14]. A recently discovered regulator of p53 activity is the type I IFN pathway. IFN-α/β stimulates transcription of the gene encoding p53, through ISRE sites present in its promoter, resulting in an early induction of the protein [4], which is later held in time by the action of ARF [15]. Importantly, type I IFN seems also to induce p53 protein stabilization through posttranslational mechanisms that involve STAT1-dependent transcriptional downregulation of Mdm2 and direct protein interaction with p53, at least upon DNA damage [16]. In addition, although acute IFN treatment does not cause the activation of p53 [4], chronic treatment of primary cells with IFN-β leads to p53 phosphorylation at Ser-15 and engagement of p53 downstream genes, indicative of p53 activation [17]; suggesting that the cellular response to IFN-β is time-dependent. Infection with some viral agents, such as vesicular stomatitis virus (VSV), Newcastle disease virus (NDV) or herpes simplex virus (HSV), probably through the production of interferon, boosts p53 expression, and induces the phosphorylation of p53 at Ser-15 by ataxia telangiectasia mutated (ATM) kinase [4]. Phosphorylation of p53 in other amino acid residues in response to IFN or virus infection cannot be excluded since p53 has been also identified as a substrate for the double-stranded RNA (dsRNA) activated protein kinase R (PKR), an IFN-inducible protein kinase which can induce the phosphorylation of human p53 at Ser-392 *in vitro* [18,19], an event that has been proposed to be related with the stabilization and activation of p53 [20–22]. In this regard, induction of p53 as a downstream event of tumor necrosis factor alpha (TNF-α)-induced upregulation of PKR has been reported, and impaired p53-mediated responses to doxorubicin in PKR KO cells have been described [17,18,23].

## 3. p53 regulation of apoptosis in the context of viral replication

p53 induces the expression of a wide number of genes that ultimately impose a state of G1-phase cell cycle arrest or apoptosis. Previous studies indicate that the role of p53 in the control of virus infection depends at least to some extent on its ability to activate apoptosis through the transactivation of p53 classic target genes such as the members of the BH3-only family of proteins PUMA and Noxa [4,24]. Interestingly, a recent study also indicates that the type I IFN-inducible protein kinase PKR, is a novel p53 target gene, suggesting that PKR could also play a role in p53-dependent induction of apoptosis upon viral infection [25].

An intriguing unresolved question is whether TNF-related cytokines also contribute to p53-dependent apoptosis in response to viral infection. TNF-α is expressed at high levels upon infection with several viruses such as influenza A, human immunodeficiency virus (HIV) and Ebola virus (EBOV) [26–28]. Expression of TNF-α by virus-infected cells induces maturation of resident dendritic cells (DCs) through upregulation of the costimulatory molecules CD80 and CD86 [29]. Besides, TNF-α synergizes with type I IFNs and polarizes the adaptive immune response towards a Th1 type (cellular immunity) [30,31]. Moreover, recent reports indicate that TNFR-mediated apoptosis of virus-infected cells may be a key component at the interface between innate and adaptive immunity by allowing DCs to cross-present viral antigens to naïve T cells [32]. Specifically, the p53 direct target gene TRAIL-R2/DR5/KILLER has been previously shown to promote DC maturation and immune enhancement *in vivo* by activation of caspase 8-dependent apoptosis [33]. Viral antigens produced by apoptotic virus-infected cells are then engulfed by DCs which have the ability to present them to CD8^+^ T cells through major histocompatibility complex (MHC) class I [34]. This process may be especially important in triggering immunity against viruses that do not naturally infect DCs, and includes not only TRAIL-R2/DR5/KILLER but also other p53 direct target genes such as Fas/Apo1/CD95 [35,36]. Of note, activation of p53 downstream targets that specifically mediate its proapoptotic activity in response to virus infection may be also cell type specific. Thus, although VSV infection induced increased apoptosis in a p53 dose-dependent fashion in mouse embryonic fibroblasts (MEFs) [5], infection of HCT116 tumor cells retaining wt p53 with VSV, did not elicit an apoptotic response [37]. These findings also indicate, that virus-induced p53-dependent apoptosis may require the activity of p53 downstream target genes that could be impaired in cancer cell lines such as HCT116.

p53-dependent apoptosis has been demonstrated as a useful mechanism to control some virus infection, as shown for VSV [5,6,38], influenza A virus [7], herpes simplex virus (HSV) [8], or poliovirus [9]. In sharp contrast other viruses have evolved mechanisms to use p53 activity in its own benefit. Thus, p53 enhances the ability of human cytomegalovirus (HCMV) to replicate in fibroblasts [39], increases respiratory syncytial virus (RSV) [40] or adenovirus replication [41] and its absence has a detrimental effect in the growth of encephalomyocarditis virus (EMCV) and parainfluenza virus [38]. A possible explanation for this striking observation is that, while early apoptosis is probably detrimental for replication of some viruses, other viruses may benefit from apoptosis late in the replication cycle in order to improve transmission of newly-formed viral particles to other cells or hosts [42].

The complexity of the p53 response to viral infection is supported by studies addressing the role of p53 on dsRNA-induced apoptosis. Although p53 is often considered as a cell death inducer, there are also cell types and situations where the presence of p53 prevents cell death by inducing cell cycle arrest. Thus, the induction of G1 arrest mediated by p53 in response to dsRNA has been suggested as a putative mechanism to delay apoptosis and consequently increase EMCV replication [38]. Moreover, p53 functions as a transcription factor are also subverted by some viruses in their own benefit. Thus, p53 activates the transcription of the HCMV L44 protein, required for virus replication, and there are 21 exact matches for the p53 binding site within the virus genome, including sites that could influence the expression of proteins involved both in the structure and replication of HCMV [39]. p53 also cooperates with E1A to enhance transcription from the major late adenovirus promoter, triggering the adenovirus lytic cycle and increasing virus yield [41]. Finally, p53-dependent downregulation of IL-6 has been suggested to mediate the positive effect of p53 on RSV replication. However, the decrease in the p53 levels induced by RSV to delay apoptosis paradoxically enhances the host inflammation response that hampers virus replication [40].

## 4. p53 direct target genes in the type I interferon pathway

Takaoka and colleagues [4] identified p53 as a type I IFN transcriptional target and demonstrated the existence of a crosstalk between p53 and the IFN pathway both in tumor suppression and antiviral defense [4]. An increasing body of work indicates that in fact, p53 crosstalk takes place both at the level of IFN production and IFN signaling. These findings indicate that p53, through direct transcriptional upregulation of several target genes, influences the type I IFN pathway that further suggests an important role for p53 in innate immunity. Here, we have summarized a list of genes that are transcriptional targets of both type I IFN and p53 and the possible implications of this crosstalk in antiviral immunity.

### 4.1. IRF9

IRF9 is, along with STAT1 and STAT2 [43], an integral part of the ISGF3 complex, with a critical role in the activation of antiviral genes, as demonstrated by the severely immuno-compromised status of *Irf9*–/– mice [44]. The ISGF3 complex regulates the expression of antiviral genes harboring ISREs in their promoters, including p53 [4]. However, IRF9 is also a p53 transcriptional target. We have recently shown that p53 induces the transcriptional upregulation of IRF9, which contributes to enhance the IFN signaling pathway leading to upregulation of ISRE-dependent genes such as IRF7, retinoic acid-inducible gene 1 (RIG-I) or MxA [6]. Importantly, and probably due to IRF9-dependent activation of IRF7, p53 also contributed to IFN production in cells infected with viruses such as Sendai (SeV), (Cantell strain) [6]. This link between p53 activity and production of IFN by infected cells has been also described during HIV infection, suggesting that this effect is not dependent on the virus type [45]. Consistent with an important role of p53 at enforcing the type I IFN signaling pathway, mice that conserve wt p53 were more protected from SeV infection than their p53–/– counterparts [6]. Similar to our results showing upregulation of IRF9 by p53 at transcriptional level, Shen and colleagues reported that upregulation of p53 induced in response to influenza virus infection resulted also in the transactivation of IRF9 [46]. Finally, an upregulation of IFN signaling upon Hepatitis C virus (HCV) infection mediated by direct p53-IRF9 protein interaction has been also suggested [47]. Further research is required to determine whether these cellular responses are dependent on the kinetics of infection and/or the multiplicity of infection (MOI) employed in such experiments. The recent findings by Shen and colleagues indicating that p53 levels are upregulated upon infection with influenza A virus, leading to IRF9 and Bax upregulation [46], are consistent with previous studies that demonstrated that p53 is important for influenza-induced apoptosis, and thus, cells retaining wt p53 have an enhanced apoptotic response to influenza A infection, which results in impaired viral replication [7]. These findings indicate that p53 activity contributes to both the enhancement of the type I IFN through IRF9 upregulation as well as virus-induced apoptosis in response to influenza infection. Furthermore, treatment of cancer cells harboring wt p53 with Ribavirin induced mammalian target of rapamycin (mTOR)-dependent activation of p53 that resulted in IRF9 transcriptional upregulation and enhancement of IFN signaling [48]. These studies indicate that p53 directly regulates IRF9 activity and provides a new link between the type I IFN pathway and p53, not only with implications in antiviral defense but also in tumor suppression.

### 4.2. TLR3

The first member of the TLR protein family was a *Drosophila* protein implicated in dorsoventral patterning during embryonic development [49]. Further studies indicated that the intracellular domain of *Drosophila* Toll showed striking similarities to the intracellular domain of the mammalian interleukin-1 (IL-1) receptor [50]. To date different human homologous of *Drosophila* Toll have been identified and shown to induce activation of several pro-inflammatory cytokines upon pathogen exposure including type I IFNs, TNF-α and IL-8 [51]. TLRs are essential components of innate immunity due to their ability to sense pathogen-associated molecular patterns (PAMPs). Thus, TLRs along with other cellular pathogen sensors such as RIG-I or the NOD-like receptor (NLRs) family of proteins are considered pattern recognition receptors (PRRs) [52]. TLRs are well conserved from mammals to invertebrates underscoring their importance in innate immunity. In mammals, TLRs are composed presumably by 13 family members [53]. Among TLRs, TLR3, TLR7 and TLR9 play a major role in the recognition of virus infection due to their ability to sense double-stranded RNA (dsRNA), single stranded RNA (ssRNA) and CpG DNA motifs respectively [53], which are main subproducts of viral replication [54]. TLR3 is expressed in most cell types even though it might play a major role in recognition of dsRNA by conventional DCs, where it is expressed in cellular endosomes as opposed to fibroblasts in which it is expressed in the cell surface [55]. Upon recognition of dsRNA, TLR3 induces IFN production as well as IL-8, RANTES, and TNF-α, leading to DC maturation which enhances their ability to present virus-derived antigens to naïve T cells by exposure of such antigens by class I and II histocompatibility complex (MHC-I and MHC-II) [56]. Therefore, TLR3 plays a major role at the interface between innate and adaptive immunity.

A recent paper by Taura *et al*. indicated that TLR3 is a direct p53 downstream target gene [57]. Interestingly, the authors found not only a functional p53 binding site in the TLR3 promoter, but also they showed that TLR3-dependent up-regulation of IL-8 in response to dsRNA, is dependent to a great extent on p53 in the p53 wt HCT116 human colon cancer cell line [57]. Moreover, TLR3 stimulation with synthetic dsRNA (poly I:C) in wt p53 HCT116 cells induced phosphorylation of the TLR3 downstream target IRF3, a key event for the transactivation of the IFN-β promoter, whereas IRF3 phosphorylation was severely impaired in p53–/– cells [57]. In agreement with the author’s findings, unpublished results from our group indicate that cells lacking p53 are in fact, severely impaired in their ability to activate the IFN-β promoter. A still open question is whether p53 affects the function of conventional DCs, which express high levels of TLR3. If so, such findings would imply that p53 functions are important not only for innate antiviral immunity, but also to trigger adaptive immunity. Indeed, recent findings indicating that the type I IFN response is important for DC activation and antigen presentation [58,59] suggest that this may be actually the case. Moreover, this could also explain why p53–/– mice are more sensitive to some viral infections [4–6]. Further research is required to evaluate the possible role of p53 in the interface between innate and adaptive immunity as well as other possible cross-talks between p53 and the TLR family.

### 4.3. IRF5

IRF family members have been identified as transcription factors that act to prevent different types of cell stress, including viral infection and DNA damage [60]. In fact, the first IRF member discovered, IRF1, was previously shown to cooperate with p53 in the induction of apoptosis in cells undergoing DNA damage, as well as in the induction of cell cycle arrest through p21 [61]. Three members of the IRF family of proteins seem to play a major role as direct activators of the type I IFN response upon viral infection, IRF3, IRF7 and IRF5 [60,62]. These proteins are phosphorylated and translocated to the nucleus upon recognition of viral PAMPs by TLRs where they form part of a multimeric protein complex containing not only the IRFs, but also ATF-2/c-Jun, and NFkappaB, which leads to transactivation of the IFN-β promoter, namely the type I IFN enhanceosome [63].

IRF5 directly interacts with the TLR adaptor protein MyD88 and regulates TLR-dependent expression of pro-inflammatory cytokines such as IL-6 and IL-12 [64]. Interestingly, as opposed to IRF3 and IRF7, IRF5 activity over the type I IFN promoters seems to be to some extent virus-specific. IRF5 activity is upregulated in cells infected with NDV rather than in SeV infected cells [64]. These findings could indicate that IRF5 activity is restricted to viruses that are sensed by TLRs, and not to viruses preferentially recognized by the RIG-I signaling pathway, which seems to be the case of SeV [65]. Another unique feature of IRF5, as opposed to IRF3 and IRF7, is that its expression seems to be restricted to certain types of tissues. Constitutive expression of IRF5 can be detected primarily in lymphoid tissues and peripheral blood leukocytes (PBLs), and low levels of IRF5 can be found in skeletal muscle and prostate [66]. In agreement with the idea that IRF5 function is tissue restricted, MEFs from *Irf5*–/– mice did not show any defects in their ability to produce type I IFN when infected *in vitro* with VSV or herpes simplex virus-1 (HSV-1) but these mice were more susceptible to VSV and HSV-1-induced disease *in vivo* [67].

IRF5 was identified as a direct p53 target gene by Mori and colleagues [68]. In this study the authors noticed an increase in IRF5 levels in cancer cell lines retaining wt p53 exposed to DNA damaging agents, and confirmed that p53 was able to bind to and transactivate the IRF5 promoter [68]. More recent studies by Yanai and colleagues indicate that, in comparison with IRF1 that cooperates with p53 in both the induction of cell cycle arrest and apoptosis in response to DNA damage [61], IRF5 cooperates with p53 only in the induction of apoptosis in response to DNA damage but not in cell cycle arrest [67]. Even though both IRF5 and p53 seem to play an important role in innate immunity, it is currently unknown whether these two proteins cooperate in the induction of type I IFNs and/or antiviral genes.

### 4.4. ISG15

ISG15 is an ubiquitin homolog that regulates the function of multiple IFN-inducible genes upon activation of the type I IFN pathway [69]. Discovered more than two decades ago, its function as an ubiquitin-like protein modifier (a process that has been termed ISGylation) it’s just starting to be understood [69,70]. ISG15-dependent ISGylated proteins include important antiviral proteins such as PKR, RIG-I, or antiviral effector proteins such as MxA [71]. ISGylation usually drives protein activation since, as opposed to ubiquitylation; ISGylation does not promote protein degradation [69]. Thus, ISG15 impedes virus-dependent degradation of IRF3, prompting IFN-β transactivation [72]. However, the specific mechanisms by which ISG15 promotes the activation of its target genes are in most of the cases largely unknown [69].

The importance of ISG15 in the antiviral immune response is underscored by the fact that is one of the earliest IFN-induced genes [73]. Moreover, ISG15 has been shown to participate in specific aspects of antiviral immunity such as inhibition of EBOV budding and HIV assembly [74,75] and ISGylation by ISG15 has been also shown to be inhibited by influenza B non structural protein 1 (NS1) [76].

ISG15 was identified as a p53 direct target gene by Hummer and colleagues in 2001 [77]. The authors reported the presence of a functional p53 binding site adjacent to the core ISRE site of ISG15 [77]. Intriguingly, this paper suggests that p53 promotes ISG15 upregulation after dsRNA stimulation rather than in response to IFN treatment or virus infection [77]. In view of the recent findings that link p53 and TLR3, it is tempting to speculate that the observed effects on ISG15 could be mediated through p53-dependent upregulation of TLR3 activity. Further investigations are required to decipher whether ISG15 may participate in the p53-dependent antiviral response and whether p53 may induce a different panel of genes in response to virus infection or dsRNA treatment.

## 5. p53 as a target of viral antagonism

Upon virus infection, IFN and the tumor suppressor p53 are employed by host cells as components of their antiviral defense mechanisms. Thus, viruses need to tightly oppose these antiviral surveillance mechanisms of the host. Viruses have evolved elaborate mechanisms to subvert p53-mediated host innate immune responses. Either p53 itself or cellular factors involved in its downstream activities are inactivated by various viral proteins either by releasing cells from cell-cycle checkpoints or by protecting cells from p53-dependent apoptosis [78–80]. The number of viruses that have been shown to interfere with p53 activity has increased during the last years. Simian virus 40 (SV40) large T antigen [78,81]; Epstein Barr virus (EBV) BZLF1 [82] and EBV EBNA3C [83]; Kaposi’s sarcoma associated herpesvirus (KSHV) LANA, LANA2 and ORF K8 [84–86]; the Human herpesvirus 6 (HHV6) ORF1 [87], all interact with p53 and inhibit its activity. Adenovirus E1B-55K and E4-ORF6 proteins, human papillomavirus (HPV) E6, EBV EBNA-5, KSHV vIRF4 and the vaccinia virus (VV) B1R kinase, all induce the degradation of p53 [88–93]. Other proteins such as Adenovirus E1A or HPV E7 stabilize p53 but inhibit its transcriptional activity [94–98]. KSHV vIRF1 interacts with the ATM kinase to block its activity, thereby reducing p53 phosphorylation at the serine 15 residue and increasing p53 ubiquitylation [99,100]. EBNA-5 and polyoma virus target and inhibit critical ARF-mediated signals for p53 activation [101,102]. Other example such as the hepatitis B virus (HBV) X protein has been shown to interact with p53 and inhibits its functional activity in multiple ways [103].

As mentioned earlier, the number of viruses that has been demonstrated to control apoptosis induced by a p53-dependent mechanism has increased in the latest years, however not all the viruses induce p53 downregulation or block apoptosis induced by p53-dependent pathways. Some studies have demonstrated how some viruses induce an increase in p53 levels or activation of p53-regulated pathways. Thus, p53 activation by EBV LMP1, cytomegalovirus (CMV) IE84 or IE72, RSV fusion protein F or HIV-1 [104–107] and both upregulation and activation of p53 by KSHV viral cyclin [108] have been reported.

## 6. Summary

**Figure 1.**
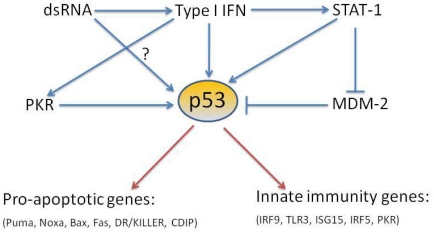
Schematic of the activators and effectors of p53-dependent response in antiviral immunity.

p53 is a tumor suppressor gene which is found mutated or absent in more than 50% of human cancers [109]. In fact, due to the ability of p53 to respond in many ways to DNA damaging insults, this protein has been called ‘the guardian of the genome’ [110]. However, p53 is also present and functional in organisms whose life span precludes them from undergoing cancer-related diseases such as flies and worms [111,112]. Moreover, a recent body of work indicates that besides ‘classic’ p53 inducers such as DNA damage and oncogene expression, its function can be also promoted by hypoxia, nutrient deprivation and viral infection [4,113,114]. These findings, and the recently identified functions of p53 in both virus-induced apoptosis and upregulation of the type I IFN response, are summarized in Figure 1, and suggest, that p53 may act as a virus induced cellular stress sensor. The role of p53 in antiviral immunity may help to explain to some extent why this protein is so highly conserved in evolution.
